# A Spotty Liver of Pregnancy

**DOI:** 10.1177/2324709614551558

**Published:** 2014-09-26

**Authors:** Meagan Gray, Don C. Rockey

**Affiliations:** 1 Medical University of South Carolina, Charleston, SC, USA

**Keywords:** hepatitis, herpes simplex, acute liver injury, pregnancy

## Abstract

Herpes simplex virus (HSV) hepatitis by definition constitutes disseminated herpes simplex infection; it is rare, with only approximately 130 cases reported in the literature. Although HSV hepatitis typically occurs in immunocompromised hosts, pregnancy—especially the third trimester, has been identified as a risk factor for its development. This is likely because of the fact that humoral and cell-mediated immunity decrease throughout pregnancy and nadir in the third trimester with decreased T-cell counts and altered B/T lymphocyte ratios. Here, we report on a patient with HSV 2 hepatitis in a previously healthy 27-year-old woman in her 23rd week of pregnancy. She initially presented with nausea, vomiting, and abdominal pain and was found to have acute hepatocellular liver injury and a systemic inflammatory response syndrome. Broad-spectrum antibiotics and acyclovir were promptly initiated. Liver biopsy, serum DNA polymerase chain reaction (PCR) as well as a labial ulcer culture and PCR were all positive for HSV 2. The patient recovered completely; however, her fetus did not survive. Review of the literature emphasizes that presentation with disseminated HSV infection typically occurs in the third trimester of pregnancy. This report emphasizes that abdominal pain combined with fever and hepatic dysfunction in pregnancy should prompt immediate consideration of the diagnosis of HSV hepatitis. Furthermore, given the high mortality rate and effective treatment, empiric treatment with acyclovir should be considered early in all potential cases.

## Case Presentation

A 27-year-old woman, gravida 2, para 1 (23 weeks), presented with a 1-day history of fever, epigastric pain, nausea, vomiting, and diarrhea. There was no melena, hematochezia, or hematemesis. She had a history of a previous full-term uncomplicated pregnancy. She was receiving recommended prenatal care. Medications included cefuroxime (prescribed 4 days prior for a urinary tract infection), ondansetron, ferrous sulfate, and a prenatal vitamin. She denied use of alcohol, acetaminophen, or herbal medications. She denied contractions, vaginal bleeding, or fluid loss. On examination, she appeared to be in distress. Blood pressure, temperature, and heart rate were 105/55 mm Hg, 100.7°F, and 124 beats/min, respectively. Her abdomen was soft but very tender to palpation in the epigastrium and right upper quadrant. She had diminished bowel sounds without rebound or guarding. There was no scleral icterus. Fetal heart tones were normal.

Laboratory data revealed a white blood cell count of 2040/cm^3^ (absolute neutrophil count 1410/cm^3^), hemoglobin 8.1 g/dL, hematocrit 24.9%, and platelet count 145 000/cm^3^. Liver tests revealed a total bilirubin 0.5 mg/dL, aspartate aminotransferase (AST) 701 IU/L, alanine aminotransferase (ALT) 284 IU/L, and alkaline phosphatase 103 IU/L. The international normalized ratio (INR) was 1.16. The blood urea nitrogen was <3 mg/dL and creatinine 0.5 g/dL. Chest radiograph was negative. *Right* upper quadrant ultrasound revealed a normal biliary system. Intravenous vancomycin, piperacillin–tazobactam, and acyclovir were initiated empirically.

The patient’s neurological status remained normal. Repeat laboratory data 24 hours after presentation revealed total bilirubin 0.7 mg/dL, AST 1902 IU/L, ALT 731 IU/L, and alkaline phosphatase 133 IU/L. The INR was 1.33, factor V 110%, and fibrinogen 313 mg/dL (normal 250-400 mg/dL). A peripheral smear showed no evidence of hemolysis and repeat platelet count was 120 000/cm.

Further laboratory evaluation revealed no acetaminophen in the blood. Complete hepatitis A, B, C, and E serologies were all negative. Anti-nuclear antibody screen and anti-double-stranded DNA antibody were negative. Lactate was normal.

**Figure 1.**
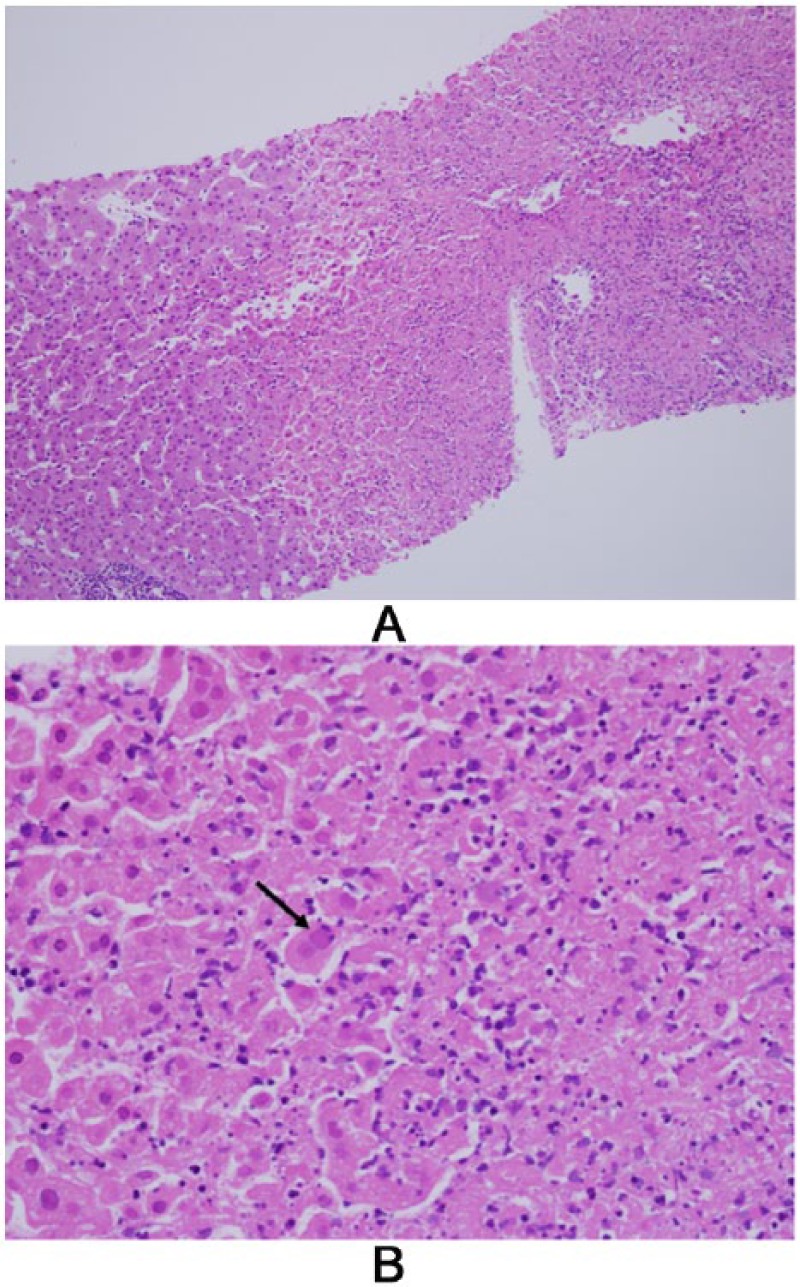
Hepatic histology (hematoxylin and eosin). In (A) is shown a low magnification image of hematoxylin and eosin–stained liver; shown on the left is an area with mostly normal hepatocytes, while in the center and on the right is a large area of necrotic hepatocytes, with extensive debris and some inflammatory cell infiltration. In (B) is shown a high magnification image of hematoxylin and eosin–stained liver; several nuclei with intranuclear inclusions, and a characteristic (of herpes simplex virus) “ground glass nucleus” with margination of the chromatin are shown, a classic example of a ground glass nucleus is shown in the center (arrow). Images provided by David Lewin, MD (Medical University of South Carolina).

**Figure 2.**
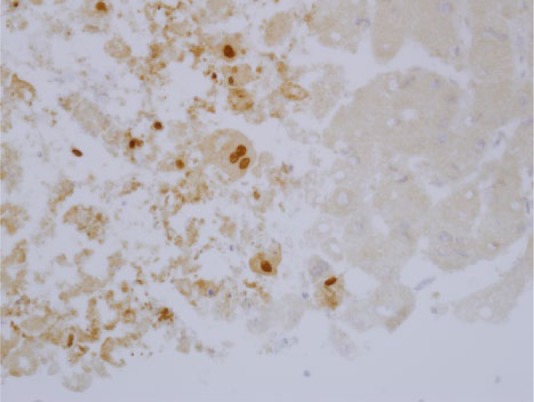
Immunohistochemistry. The liver specimen shown in Figure 1 was fixed and labeled with anti-HSV-2 antibody. Brown nuclei, containing HSV 2, are shown. Image provided by David Lewin, MD (Medical University of South Carolina).

The patient’s abdominal pain continued to worsen and she developed what was perceived to be an acute abdomen with diffuse peritonitis. Therefore, she was taken to the operating room for diagnostic laparoscopy. Although no pathology was found to explain the peritonitis, the patient was noted to have an abnormal, “spotty” or mottled appearing liver, and a liver biopsy was performed. Liver histology revealed multifocal geographic areas of necrosis with associated neutrophils (Figure 1A). Within the necrotic areas, the nuclei had intranuclear inclusions, ground glass change with margination of the chromatin, and multinucleation (Figure 1B). There was no evidence of fat, and immunohistochemical stains for HSV 2 were positive (Figure 2). An ulcerated lesion from the right labia minora was identified and a culture and viral polymerase chain reaction (PCR) revealed HSV 2. HSV 2 IgG was positive and HSV 2 DNA was also detected in the serum. Cytomegalovirus and Epstein–Barr virus DNA plasma viral loads were negative. HIV 1 and 2 antibodies were negative as was an HIV RNA viral load.

The patient was continued on intravenous acyclovir and antibiotics were discontinued. Her clinical course was subsequently complicated by chorioamnionitis secondary to preterm premature rupture of membranes, which resulted in loss of the fetus during the 24th week of pregnancy, but the patient recovered completely with normalization of liver tests.

## Discussion

Herpes simplex virus hepatitis constitutes a disseminated herpes simplex infection, and is rare, with approximately 130 cases reported in the literature,^1^ including in 27 patients occurring during pregnancy.^2^ The mechanism underlying this apparent predisposition^3^ appears to be related to the fact that the third trimester of pregnancy is associated with a nadir in humoral and cell-mediated immunity with decreased T-cell counts and altered B/T lymphocyte ratios.^4,5^

The differential diagnosis of abdominal symptoms in the second half of pregnancy includes premature labor, placental abruption, intra-amniotic infection and the less likely uterine rupture, aortic dissection, spontaneous hemoperitoneum, and uterine incarceration. Common causes of acute abdominal pain in adults should also be considered including acute gastroenteritis, cholecystitis, cholangitis, pancreatitis, hepatitis from any cause, bowel obstruction, lower lobe pneumonia, peptic ulcer disease/perforated ulcer, and the much less likely adrenal hemorrhage. Additionally, pregnancy-related liver diseases such as HELLP (hemolysis, elevated liver enzymes, and low platelets) syndrome and AFLP (acute fatty liver of pregnancy) must also be considered.

At presentation, the patient’s fever and tachycardia placed bacterial infection high in the differential diagnosis. While proceeding with the workup, she was started on broad-spectrum antibiotics and aggressively hydrated. Hypotension in pregnancy should be initially managed with volume resuscitation using intravenous fluids and placing patient in the left lateral decubitus position to prevent compression of the inferior vena cava by the gravid uterus. In the case of septic shock, vasopressor therapy should be added early. First-line therapy in pregnancy is norepinephrine followed by phenylephrine, although these agents vasoconstrict uterine blood vessels and reduce fetal blood flow.^6^

Imaging to evaluate abdominal pain is complicated in pregnancy because of the risk of radiation injury to the fetus. Ultrasound is the preferred imaging modality followed by magnetic resonance when ultrasound is equivocal.^7,8^ Magnetic resonance is preferable to computed tomography because it avoids fetal radiation exposure. Chest and abdominal x-rays are safe for pregnant patients since the radiation exposure is considered to be too low to cause adverse effects on the fetus.^9^

The data provide a definitive diagnosis of disseminated HSV; the initial infection likely sexually transmitted. Given the active ulcerated lesion on her labia minora at the time of the presentation, we believe that this was a primary HSV 2 infection as opposed to reactivation from a prior infection. Her course was unusual in that she developed apparent peritonitis, the cause of which was not clear. We speculate that perhaps irritation of the liver capsule caused severe pain, and may have mimicked peritonitis. Notwithstanding, laparoscopy can be performed during any trimester. In nonurgent situations, the second trimester is the safest—there is risk of spontaneous miscarriage during the first trimester and the procedure is technically difficult in the third trimester because of the enlarged uterus.^10^ Laparotomy is also safe in pregnancy, and patients undergoing laparoscopy and laparotomy have been shown to have similar outcomes. Laparotomy is preferred in cases of hemodynamic instability and emergent situations.^11^ Common nonobstetric conditions requiring surgery during pregnancy are appendicitis, gallstone disease, ovarian disorders (torsion, neoplasm), trauma, and bowel obstruction.

Liver diseases specific to pregnancy must always be considered when pregnant patients present with abnormal liver tests. Each has a relatively unique timing and pattern of injury, and thus can largely be differentiated from one another based on clinical and laboratory features (Table 1). Hyperemesis gravidarum occurs in the first trimester of pregnancy with persistent vomiting that may be severe enough to cause dehydration, electrolyte imbalances; the cause of elevated liver tests in this disorder is unknown. Treatment is supportive with antiemetics and intravenous fluids. Intrahepatic cholestasis of pregnancy occurs in the second and third trimesters of pregnancy, presumably because of the cholestatic effects of pregnancy-related hormones. Pruritus, worse at night, and jaundice, are typical. Aminotransferases may be substantially elevated (2-10 times above the upper limit of normal). Diagnosis is confirmed by finding elevated fasting serum bile acids. Ursodeoxycholic acid is first-line therapy.^12^

**Table 1.** Patterns of Liver Injury in Pregnancy.

Disease	Trimester	Aminotransferases	Bilirubin	INR	Treatment	Prognosis*
Hyperemesis gravidarum	First	Up to 2-3 times upper limit of normal	<4	Always normal	Supportive	Excellent
Intrahepatic cholestasis of pregnancy	Second/third	Elevated sometimes rarely >1000	Elevated but <6	Typically normal	Ursodeoxycholic acid	Good
AFLP	Third	Elevated usually <1000	Elevated	Usually elevated	Delivery	Guarded
HELLP	Third	Elevated, variable, 5-20 times upper limit of normal	>1.2	Sometimes elevated	IV magnesium, BP control, delivery	Guarded
Acetaminophen	Any	>50 times upper limit of normal	Elevated usually <10	Usually elevated	NAC	Good if NAC administered promptly
Ischemia	Any	>50 times upper limit of normal	Elevated usually <4	Usually elevated (<3)	Supportive	Variable, depends on underlying disease
Viral	Any	>25 times upper limit of normal	Elevated	Variable	Supportive	Variable
Drug-induced	Any	>25 times upper limit of normal	Elevated	Variable	Supportive	Variable
Autoimmune	Any	5-10 times upper limit of normal	Normal	Variable	Corticosteroids, azathioprine	Variable

* Prognosis assignments are estimates only. Abbreviations: INR, international normalized ratio; AFLP, acute fatty liver of pregnancy; HELLP, hemolysis, elevated liver enzymes, and low platelets; IV, intravenous; BP, blood pressure; NAC, *N*-acetylcysteine.

Acute fatty liver of pregnancy and HELLP syndrome are 2 medical and obstetric emergencies that typically occur during the third trimester. AFLP presents with nausea, vomiting, abdominal pain, and jaundice. Laboratory findings are remarkable for hyperbilirubinemia, hypoglycemia, leukocytosis, elevated creatinine, and often disseminated intravascular coagulation. Elevation in aminotransferases is typically minimal. Diagnosis is made with a careful history and physical exam in combination with typical findings (exclusion of other disorders, in particular negative viral serologies). Immediate recognition of AFLP is essential since treatment consists of prompt delivery of the fetus. Maternal and fetal mortality approach 18% and 23%, respectively. The mother’s condition usually improves within 48 to 72 hours of delivery.^13,14^ HELLP syndrome is a variant of severe preeclampsia (hypertension and proteinuria) diagnosed by either Tennessee or Mississippi classification systems with varying limits for thrombocytopenia, elevated AST and elevated lactate dehydrogenase. Similar to AFLP, HELLP can be complicated by disseminated intravascular coagulation and renal failure. Bed rest with strict blood pressure control (<155/100) and intravenous magnesium should be initiated immediately. The age of the fetus and medical complications determine the timing of the delivery. Maternal and fetal mortality are 5% and 30%, respectively.^15^

Liver diseases not unique to pregnancy can also present at any time in expectant mothers. Acute viral hepatitides (A, B, C, D, and E) typically present with evidence of hepatocellular injury, most often out of proportion to evidence of biliary injury. Hepatitis A and B are typically self-limited and managed supportively. In rare instances of acute hepatic failure or protracted severe hepatitis from acute hepatitis B, antiviral therapy may be used.^16^ Lamivudine has been used safely during pregnancy^17^ as have telbivudine^18^ and tenofovir. Hepatitis B virus can be transmitted vertically from mother to fetus, and thus, lowering high serum hepatitis B viral loads to less than 10^6^ copies/mL has been recommended.^19^ Acute hepatitis C virus infection is most commonly asymptomatic, but can present with right upper quadrant pain and acute hepatitis, similar to this case. Hepatitis E virus is typically identified in patients who have traveled to endemic areas (Asia, Africa and/or Central America). Pregnancy confers a higher risk of fulminant hepatic failure from hepatitis E, with a high mortality rate of 15 to 25 percent in the mother. Fetal loss is a consequence of maternal death and not a direct effect of the virus.^20^ Rarely, autoimmune hepatitis presents during pregnancy. Serum antinuclear antibodies, smooth muscle antibodies, γ-globulin and p-ANCA levels are often helpful, as is liver biopsy in certain situations. Antibodies to SLA and Ro/SSA have been associated with a complicated course.^21^ The mainstay of treatment is corticosteroids with or without azathioprine. Prednisone therapy alone is preferred, although not without risk as glucocorticoids significantly increase the incidence of cleft lip and palate in the fetus.^22^ Azathioprine, although a category D drug in pregnancy, has not been linked to congenital malformations in children born to mothers treated with azathioprine for autoimmune hepatitis during pregnancy.^23,24^

Herpes simplex virus infection is associated with a multitude of infections, including not only a variety of classic primary infections but also a host of disseminated syndromes. HSV hepatitis, by definition a disseminated process, can be caused by either HSV 1 or HSV 2. This rare disease carries up to a 39% mortality rate if untreated.^1^ Although HSV hepatitis can occur in immunocompetent patients, immunocompromised adults and pregnant women are at the greatest risk. It is important to recognize that pregnancy is considered to constitute a relatively immunocompromised state; humoral and cell-mediated immunity decrease throughout pregnancy and nadir in the third trimester with decreased T-cell counts and altered B/T lymphocyte ratios. These adaptations not only prevent maternal rejection of fetal and placental tissues but also increase susceptibility to viral infection.^4,5^

Abdominal pain combined with fever and hepatic dysfunction in pregnancy should prompt immediate consideration of the diagnosis of herpes hepatitis.^25^ Laboratory analysis typically reveals elevated liver transaminases (up to 100 times the upper limit of normal), normal or mildly elevated bilirubin, leukopenia, and coagulopathy. Our patient’s pattern of liver injury, as well as her leukopenia and mild coagulopathy was consistent with this picture. The gold standard for the diagnosis of HSV hepatitis remains liver biopsy. Typical histological findings include necrosis, inflammation, and enlarged ground glass nuclei with marginalized chromatin,^26^ as in our patient (Figure 1). HSV PCR also confirms the diagnosis. Serum viral DNA load correlates with liver transaminase levels and disease severity.^27^ Intravenous acyclovir is standard first-line therapy for disseminated disease.^28^ Serial HSV PCR levels can be measured to determine the magnitude of viremia and to detect resistance.^25^ In the case of drug-resistant HSV infection, intravenous foscarnet can be considered, although it carries a significant risk of toxicity to the renal tubules and should be used only when there is high risk of mortality.^29^ In cases of acute liver failure, liver transplantation should be considered early and immediate transfer to a liver transplant center is warranted.

With increasing awareness of disseminated HSV in pregnancy, the use of empiric acyclovir has become routine. Therefore, the mortality from disseminated HSV in pregnancy has become substantially reduced. With prompt treatment, a full recovery can be expected.^28^ Fetal outcomes are variable and depend on the overall condition of the mother and whether or not transplacental transmission of the virus occurs. Transplacental HSV transmission rates can reach 50% in mothers with disseminated HSV. Approximately 90% of in utero infections are caused by HSV 2.^30^ Possible fetal outcomes include stillbirth, acquisition of (often disseminated) HSV, which may or may not cause neonatal demise, and uninfected survival.^31^ Cesarean section is strongly advisable if vaginal lesions are present at time of delivery.^32^

In summary, HSV hepatitis, despite its high potential mortality, represents one of the few treatable causes of acute liver injury (present in our patient) and even acute liver failure (not present in our patient). Thus, it should be considered early in the course of any acute hepatitis, especially in an immunocompromised patient. Most important, empiric treatment with acyclovir should be promptly considered in all potential cases of disseminated HSV.
